# A Computed Tomography–Based Fracture Prediction Model With Images of Vertebral Bones and Muscles by Employing Deep Learning: Development and Validation Study

**DOI:** 10.2196/48535

**Published:** 2024-07-12

**Authors:** Sung Hye Kong, Wonwoo Cho, Sung Bae Park, Jaegul Choo, Jung Hee Kim, Sang Wan Kim, Chan Soo Shin

**Affiliations:** 1 Department of Internal Medicine Seoul National University Bundang Hospital Seongnam Republic of Korea; 2 Kim Jaechul Graduate School of AI Korea Advanced Institute of Science and Technology Daejeon Republic of Korea; 3 Department of Neurosurgery Seoul National University Boramae Hospital Seoul Republic of Korea; 4 Department of Internal Medicine Seoul National University Hospital Seoul Republic of Korea; 5 Department of Internal Medicine Seoul National University Boramae Hospital Seoul Republic of Korea

**Keywords:** fracture, bone, bones, muscle, muscles, musculoskeletal, prediction, deep learning, prospective cohort, fracture risk assessment, predict, predictive, machine learning, develop, development, validate, validation, imaging, tomography, scanning

## Abstract

**Background:**

With the progressive increase in aging populations, the use of opportunistic computed tomography (CT) scanning is increasing, which could be a valuable method for acquiring information on both muscles and bones of aging populations.

**Objective:**

The aim of this study was to develop and externally validate opportunistic CT-based fracture prediction models by using images of vertebral bones and paravertebral muscles.

**Methods:**

The models were developed based on a retrospective longitudinal cohort study of 1214 patients with abdominal CT images between 2010 and 2019. The models were externally validated in 495 patients. The primary outcome of this study was defined as the predictive accuracy for identifying vertebral fracture events within a 5-year follow-up. The image models were developed using an attention convolutional neural network–recurrent neural network model from images of the vertebral bone and paravertebral muscles.

**Results:**

The mean ages of the patients in the development and validation sets were 73 years and 68 years, and 69.1% (839/1214) and 78.8% (390/495) of them were females, respectively. The areas under the receiver operator curve (AUROCs) for predicting vertebral fractures were superior in images of the vertebral bone and paravertebral muscles than those in the bone-only images in the external validation cohort (0.827, 95% CI 0.821-0.833 vs 0.815, 95% CI 0.806-0.824, respectively; *P*<.001). The AUROCs of these image models were higher than those of the fracture risk assessment models (0.810 for major osteoporotic risk, 0.780 for hip fracture risk). For the clinical model using age, sex, BMI, use of steroids, smoking, possible secondary osteoporosis, type 2 diabetes mellitus, HIV, hepatitis C, and renal failure, the AUROC value in the external validation cohort was 0.749 (95% CI 0.736-0.762), which was lower than that of the image model using vertebral bones and muscles (*P*<.001).

**Conclusions:**

The model using the images of the vertebral bone and paravertebral muscle showed better performance than that using the images of the bone-only or clinical variables. Opportunistic CT screening may contribute to identifying patients with a high fracture risk in the future.

## Introduction

The globally aging society has driven an increase in the incidence of fragility fractures and imposed a significant burden on health care systems, societies, and most importantly, on patients and their families [1-3]. Thus, proactively identifying patients with a high risk of fractures is vital. There are well-established methods to evaluate the risk of fractures, such as dual-energy X-ray absorptiometry (DXA) to assess bone mineral density (BMD), which is a reference standard for the diagnosis of osteoporosis [4]. However, a large proportion of patients have never undergone DXA, and 60% of the patients with major osteoporotic fractures do not receive proper treatment to reduce the risk of fractures [5].

Opportunistic computed tomography (CT) scans can be a novel approach for identifying patients with a high risk of fractures. Along with the increase in progressively aging populations, the use of opportunistic CT scanning is increasing, with over 80 million examinations performed each year in the United States [6]. Retrieval of information that can help assess the fracture risks from opportunistic CT scans does not require additional costs, time, or equipment, and data can be retrospectively acquired. Thus, it may help reduce the efforts associated with screening patients with high risks of fractures. Several studies have assessed BMD by using opportunistic CT scans [7], mainly utilizing the attenuation data of the trabecular bone of the spine [8,9].

There have been significant advances in deep learning techniques for medical image analysis, such as the convolutional neural network (CNN) method [10]. The CNN method facilitates the utilization of highly representative, data-driven image features, arranged in a layered hierarchical structure, which are effective in successfully classifying medical images. Various images have been used in CNN to classify patients with a high fracture risk [11-14]. Previous studies have primarily focused on the use of radiographic images for fracture detection, with AUROCs reported in the range of 0.73-0.80 [14,15]. However, there is scarcity of research utilizing CT images, and this is limited to bone texture analysis. Our study may fill this gap by applying CNN techniques to CT scans, which may provide a more accurate assessment of fracture risk due to the detailed and comprehensive nature of CT imaging. Further, as paravertebral muscles are among the critical contributing factors to vertebral fractures [16,17], CT could be a valuable method for acquiring information on both the muscle and vertebral bone. Nevertheless, to our knowledge, no previous study has reported using images of both the vertebral bone and muscle from CT scans by using the CNN method. Therefore, we aimed to develop and externally validate a CT-based fracture prediction model by using images of vertebral bones and muscles by employing a deep learning method. This study may help identify patients having high risk of fractures among those who undergo opportunistic CT scans for screening or other purposes.

## Methods

### Study Design and Participants

**Figure 1:**
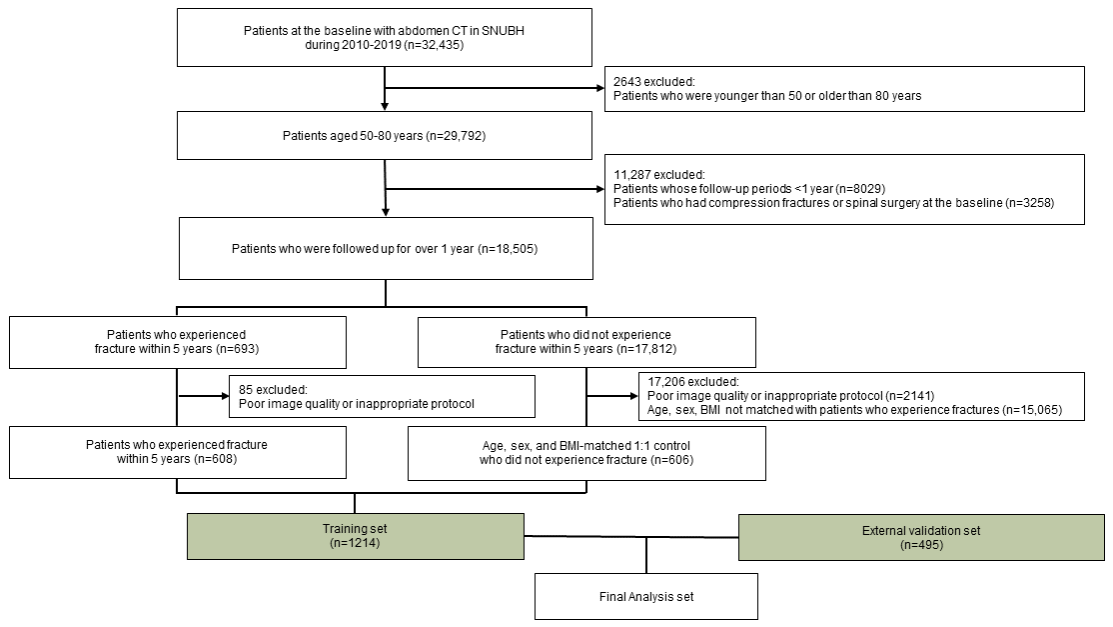
Flowchart of participant selection for this study on vertebral fracture prediction. CT: computed tomography; SNUBH: Seoul National University Bundang Hospital.

This study was based on a retrospective longitudinal cohort study of 32,435 patients having abdominal CT images at Seoul National University Bundang Hospital between 2010 and 2019. Patients who met all the inclusion criteria were included. Inclusion criteria were as follows: (1) patients who had abdominal CT imaging at Seoul National University Bundang Hospital between 2010 and 2019 and had follow-up images at the 5-year timepoint, (2) those who were aged between 50 and 80 years, and (3) those who were followed up for over a year. Further, patients who met any one of the exclusion criteria were excluded. The exclusion criteria and the number of excluded patients were as follows: (1) patients who were younger than 50 years or older than 80 years (n=2643), (2) those whose follow-up periods were less than a year (n=8029), and (3) those who had compression fractures or spinal surgery at the baseline (n=3258) (Figure 1). Finally, 18,505 patients were included in the analysis. During follow-up, 693 patients experienced vertebral fractures, while the remaining 17,812 patients did not. Among the 693 patients, after excluding 85 patients owing to the poor image quality or inappropriate CT protocols, 608 patients remained as cases.

For the control group, we selected individuals from the same time frame as the fracture cases. Among 17,812 patients who did not experience fracture, after excluding 2141 patients with poor image quality, we selected 606 age-, sex-, and BMI-matched individuals at a ratio of 1 patient to 1 control within a similar follow-up period. The fracture events were determined by reviewing medical records, with efforts to exclude any fractures associated with trauma. If patients had multiple CT scans during the follow-up, the earliest CT scan was used.

As a result, 1214 patients were eligible for analysis and constituted the development set. In addition, we developed an external validation set of 495 patients from Seoul National University Boramae Hospital by using the same protocol but without case-control matching between 2012 and 2013. An external validation set was developed to assess the performance of the intervention. The same protocol was used to ensure consistency in evaluation while allowing for a broader application of the findings in real-world settings.

### Ethics Approval

This study protocol was approved by the institutional review board of the Seoul National University Bundang Hospital (B-2104-677-402). The requirement for informed consent was waived owing to the retrospective design of this study. This study was conducted in accordance with the ethical standards laid down in the 1964 World Medical Association Declaration of Helsinki and its later amendments. This study also complies with the ethical principles for medical research.

### Primary Outcome

The primary outcome of this study was the predictive accuracy of vertebral fracture events between T12 and L4 occurring within 5 years. Vertebral fractures were defined as morphometric fractures and were confirmed using radiographic or CT images. These images were adjudicated by SHK and JHK, who were blinded to any patient information prior to their assessments. Morphometric vertebral fractures were confirmed by radiographic or reconstructed CT images with measurements of anterior (Ha), middle (Hm), and posterior (Hp) height of each vertebral body from T11 to L4 measured. Normal population was classified as having no vertebral fracture by gross visual inspection as being within normal range for vertebral height and shape. The mean (SD) of ratio of normal vertebral height was obtained from patients without incident fractures. We first calculated the anterior to posterior (Ha/Hp), middle to posterior (Hm/Hp), and posterior to posterior above and below (Hpi/Hpi+1 and Hpi/Hpi–1) ratios. Vertebral fracture was defined if any of the following ratios were more than 3 SDs below the normal mean for that vertebral level, which was 0.91 (SD 0.08), as described in previous reports [18,19].

### Measurements of Clinical Factors

Sociodemographic factors, including age, sex, and medical history, were obtained from a review of electronic medical records at baseline. Height and body weight were measured using standard methods by trained staff with a scale and wall-mounted extensometer, and the participants wore lightweight clothes. BMI was calculated as weight divided by height in meters squared (kg/m^2^). Current smokers were defined as patients who were smoking during the study period, while current alcohol consumers were defined as those who consumed 3 or more units of alcohol daily. The use of glucocorticoids was defined as using oral glucocorticoids or having been exposed to oral glucocorticoids for more than 3 months at a prednisolone dose >5 mg or its equivalent doses. Possible secondary osteoporosis is defined as osteoporosis that occurs due to factors other than primary menopause or age-related causes. It includes patients with osteoporosis and concurrent diagnosis with type 1 diabetes, osteogenesis imperfecta in adulthood, hyperthyroidism, hypogonadism, premature menopause (age<45 years), chronic malnutrition, malabsorption, or chronic liver disease [20].

### CT Protocols, Image Preprocessing, and Deep Learning Techniques

Intravenous contrast-enhanced images were obtained using CT scanners with 64 detector rows (Brilliance; Philips Medical Systems). All the patients were placed in a supine position and scanned from the diaphragm to the symphysis pubis. The reference tube current–time product was empirically set, aiming at effective radiation doses of 2 mSv. The effective tube current–time product generally ranges between 25 mA and 40 mA. The actual radiation dose was adjusted according to the body size by automatically modulating the tube current (Dose-Right; Philips Medical Systems). The values of tube voltage, collimation, rotation speed, and pitch were 120 kVp, 64 mm×0.625 mm, 0.5 seconds, and 0.891, respectively. Patients were administered 2 mL iopromide/kg (Ultravist 370; Schering) intravenously at a rate of 3 mL/s via the antecubital vein, and scanning was initiated 60 seconds after the enhancement of the descending aorta reached 150 HU. From each helical scan, the images were reconstructed using a section thickness of 5 mm.

Consecutive image processing was applied to all the CT images for accurate deep learning–based image analysis. Each axial slice of the abdominal CT scan was resampled to obtain a pixel spacing of 1×1 mm^2^. The signal intensity of each CT image was min-max normalized to the –1 to 1 range after windowing the Hounsfield unit values in the range of –200 to 1000. Subsequently, 2 classes of image data, that is, vertebral body only (bone-only) and vertebral body with paravertebral muscles (bone+muscle) were extracted from each CT image. Based on the manual annotations of the vertebral body, excluding the intervertebral disc, the vertebral body regions from T12 to L4 were extracted from the CT images, where each bone-only image had 96×96 pixels in the axial plane. Centered on the vertebral body, the images of the paravertebral muscle were automatically cropped using a rectangular box (96×144 pixels) in the axial plane (Figure S1 in Multimedia Appendix 1).

**Figure 2:**
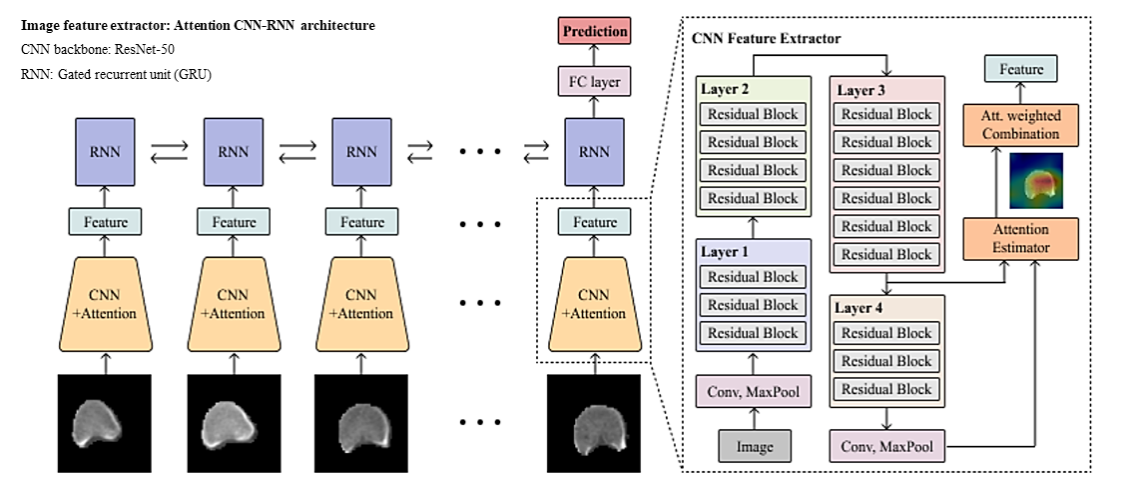
Architecture of the convolutional neural network–recurrent neural network model for vertebral fracture prediction. CNN: convolutional neural network; FC, fully connected; RNN: recurrent neural network.

Deep learning–based image features for the 5-year risk analysis of vertebral fractures were extracted using the attention CNN–recurrent neural network (CNN-RNN) model for image data (Figure 2) [21,22]. In the CNN-RNN model, ImageNet-pretrained ResNeXt-50 and gated recurrent units were employed as the CNN encoder backbone and the RNN recurrent decoder, respectively. As inputs of the model, 14 equidistant axial slices were extracted from the T12 to L4 vertebrae region of each CT image, where the starting slice was randomly selected at each training iteration for data augmentation. In the training phase, the CNN-RNN model was optimized using the Adam optimizer and cross-entropy loss, where the learning rate and batch size were 1e-5 and 128, respectively. In our image-based fracture prediction model, the utilization of CT images was primarily driven by deep learning methods, particularly CNNs. Although specific imaging parameters such as attenuation values and density were not directly used as standalone inputs, the CNN’s learning process inherently captured these aspects as part of the comprehensive image analysis. The model processed the entire CT images, extracting deep features that potentially included characteristics related to bone and muscle attenuation and density, among others. This approach allowed for a sophisticated interpretation of the CT scans, identifying nuanced patterns indicative of fracture risk. To incorporate clinical variables into our image-based prediction model, we first standardized the clinical variables to ensure consistency and comparability. Following this, we concatenated the standardized variables to the image features in the final layer of the CNN-RNN model.

**Figure 3:**
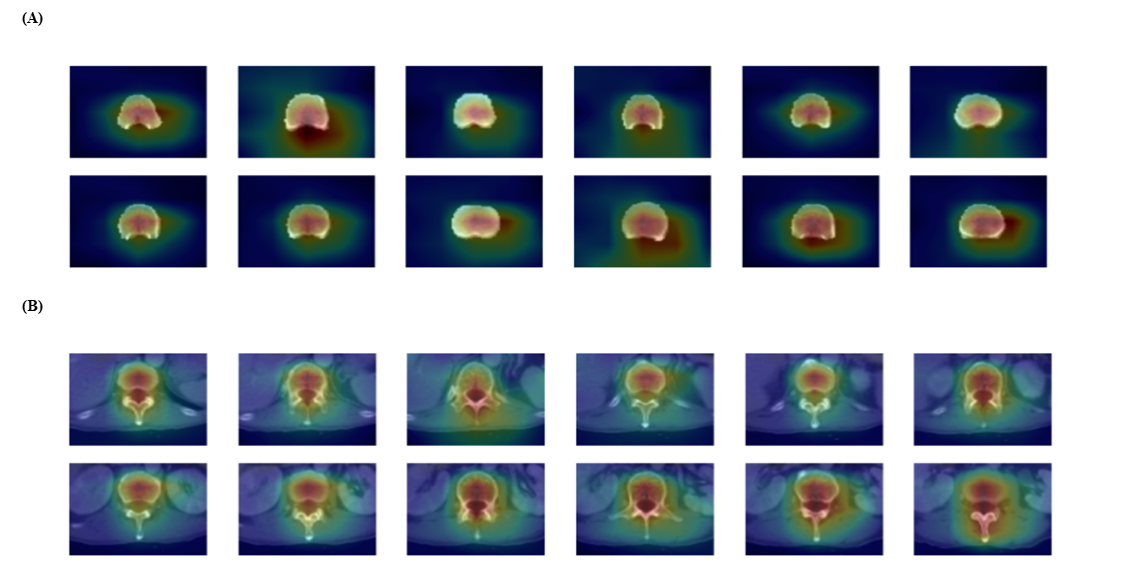
Gradient-weighted class activation mapping heatmaps of image models using (A) bone-only and (B) bone+muscle images. A heatmap was generated by gradient-weighted class activation mapping, a technique used to visualize those parts of an input image that are important for a neural network's decision, especially in convolutional neural networks.

To understand how the model identifies and differentiates key areas for predicting vertebral fractures in CT images, we employed the gradient-weighted class activation mapping technique. This approach involves highlighting the most crucial regions within the images, marked by a bright red overlay, thereby revealing the model’s decision-making process and focal areas for classification (Figure 3). The image models were developed with a high-performance computing server with 4 NVIDIA GeForce GTX 1080 Ti (NVIDIA) graphic processing units and the Ubuntu 16.04.4 operating system.

### Statistical Analyses

For the baseline characteristics, depending on the distribution, continuous parameters were presented as means with standard deviations and categorical data were presented as proportions. Comparisons between the groups having continuous variables were analyzed using 2-sided Student *t* test, whereas *χ*^2^ test was used for the categorical variables. The area under the receiver operating characteristic curve (AUROC) was calculated to compare the preprocessed images. Cases predicted to have an actual fracture event and experienced it during follow-up were defined as true positive. Those predicted to have but did not experience a fracture was designated as false positive. Cases predicted to be free from fracture events but experienced one during follow-up were defined as false negative. True-negative cases were predicted to be free of fracture events with no fractures during follow-up. Sensitivity and specificity were calculated for each time series as follows: sensitivity = true positive / (true positive + false negative) and specificity = true negative / (true negative + false positive). The risk prediction performance measures were gauged using 10-fold cross-validation.

The image-only 5-year risk analyses of vertebral fractures were conducted by applying a fully connected layer, which generates binary prediction results to the CNN-RNN feature extractor. In addition to the image-only model, clinical models (models A, B, C, and D) were developed by analyzing the corresponding clinical variables via XGBoost. The clinical variables included age, sex, BMI, use of steroids, smoking status, and possible secondary osteoporosis. Model A included age and sex as independent variables; model B additionally incorporated BMI; model C further included clinical variables such as the use of glucocorticoids, history of alcohol consumption, smoking, and possible secondary osteoporosis; and model D included type 2 diabetes mellitus, HIV, hepatitis C infection [23], and renal failure [24].

The matching of cases and controls in our study was conducted based on age and sex. This process was facilitated using the propensity score matching method, implemented through the MatchIt package in R (version 4.1.2; R Foundation). PyTorch and Scikit-learn libraries from Python were used for the analyses. A *P* value <.05 was considered significant. Correction for multiple testing was not performed across models. Statistical analyses were performed using Python (version 3.8.10; Python Software Foundation). The programs used in the experiments were PyCharm (JetBrains s.r.o.) and Visual Studio (Microsoft Corp).

## Results

### Clinical Characteristics

A total of 1709 individuals were included in the analysis. The participants were divided into a development set from Seoul National University Bundang Hospital (n=1214) and an external validation set (n=495) from Seoul National University Boramae Medical Center. As shown in Table 1, the development set was older (mean 72.5, SD 7.9 years) than the external validation set (mean 67.6, SD 8.6 years), with a statistically significant difference (*P*<.001). The proportion of females in the external validation set (390/495, 78.8%) was higher than that in the development set (839/1214, 69.1%), with this difference also being significant (*P*<.001). However, no significant differences were observed in weight and BMI between the 2 sets. When considering lifestyle factors, there was a higher prevalence of current smokers (284/1214, 23.4% vs 28/495, 5.7%; *P*<.001) and current drinkers (236/1214, 19.4% vs 34/495, 6.9%; *P*<.001) in the development set than those in the external validation set, respectively. The use of steroids was similar across both groups (*P*=.56), while the prevalence of possible secondary osteoporosis was significantly higher in the development set (122/1214, 10.1% vs 16/495, 3.2%, respectively; *P*<.001). Within 5 years of follow-up, 454 (37.4%) and 61 (12.3%) individuals experienced vertebral fractures in the development and external validation sets, respectively.

In the development set (n=1214), participants were matched based on age, sex, and BMI to compare those with incident fractures (n=608) to those without (n=606). There was a less than 1-year age difference (mean age 72.0, SD 7.6 years in the nonfracture group vs 72.9, SD 8.2 years in the fracture group; *P*<.001) and a BMI difference of less than 0.5 (mean BMI 23.7, SD 3.4 kg/m² in the nonfracture group vs 23.5, SD 3.5 kg/m² in the fracture group; *P*=.39). Gender distribution was balanced between the 2 groups (422/606, 69.6% females in the nonfracture group vs 417/608, 68.6% females in the fracture group; *P*=.96).

Despite these matched parameters, a higher prevalence of current smokers was noted in the fracture group than in the nonfracture group (169/608, 27.8% vs 115/606, 18.9%, respectively; *P*=.001) along with a significantly higher use of steroids (114/608, 18.8% vs 47/606, 7.8%, respectively; *P*<.001) and a greater prevalence of possible secondary osteoporosis (77/608, 12.7% vs 45/606, 7.4%, respectively; *P*=.001). No significant differences were observed in the height, weight, and current drinking status between the 2 groups (Table 2).

**Table 1:** Clinical characteristics of the development and external validation sets.^a^

	Development set (n=1214)	External validation set (n=495)	*P* value
Age (years), mean (SD)	72.5 (7.9)	67.6 (8.6)	<.001
Female, n (%)	839 (69.1)	390 (78.8)	<.001
Height (cm), mean (SD)	157.0 (8.4)	155.2 (7.9)	<.001
Weight (kg), mean (SD)	58.2 (9.9)	57.6 (9.7)	.22
BMI (kg/m^2^), mean (SD)	23.6 (3.5)	23.9 (3.6)	.18
Current smoker, n (%)	284 (23.4)	28 (5.7)	<.001
Current drinker, n (%)	236 (19.4)	34 (6.9)	<.001
Use of steroids,^b^ n (%)	161 (13.3)	57 (11.5)	.56
Possible secondary osteoporosis,^c^ n (%)	122 (10.1)	16 (3.2)	<.001
Vertebral fracture within 5 years, n (%)	608 (50)	61 (12.3)	<.001

^a^The variables between the groups were compared using the 2-sided Student *t* test for continuous variables and the *χ*^2^ test for categorical variables.

^b^Use of steroids was defined as the use of prednisolone 5 mg daily or equivalent over 3 months.

^c^Possible secondary osteoporosis includes patients with osteoporosis and concurrent diagnosis with type 1 diabetes, osteogenesis imperfecta in adulthood, hyperthyroidism, hypogonadism, premature menopause (<45 years), chronic malnutrition, malabsorption, and chronic liver disease.

**Table 2:** Baseline clinical characteristics of the development set comparing incident fracture groups.^a^

	Incident fracture (-) (n=606)	Incident fracture (+) (n=608)	*P* value
Age (years), mean (SD)	72.0 (7.6)	72.9 (8.2)	<.001
Females, n (%)	422 (69.6)	417 (68.6)	.96
Height (cm), mean (SD)	156.9 (8.5)	157.1 (8.3)	.32
Weight (kg), mean (SD)	58.29 (9.5)	58.11 (10.4)	.23
BMI (kg/m^2^), mean (SD)	23.7 (3.4)	23.5 (3.5)	.39
Current smoker, n (%)	115 (18.9)	169 (27.8)	.001
Current drinker, n (%)	113 (18.7)	123 (20.2)	.92
Use of steroids,^b^ n (%)	47 (7.8)	114 (18.8)	<.001
Possible secondary osteoporosis,^c^ n (%)	45 (7.4)	77 (12.7)	.001

^a^The variables between the groups were compared using the 2-sided Student *t* test for continuous variables and the *χ*^2^ test for categorical variables. Fracture (-) and (+) groups represent participants who did not and did experience fractures at 5 years of follow-up, respectively.

^b^Use of steroids was defined as the use of prednisolone 5 mg daily or equivalent over 3 months.

^c^Possible secondary osteoporosis includes patients with osteoporosis and concurrent diagnosis with type 1 diabetes, osteogenesis imperfecta in adulthood, hyperthyroidism, hypogonadism, or premature menopause (<45 years), chronic malnutrition, malabsorption, and chronic liver disease.

### Comparisons Between the Performances of Image Models in Predicting Vertebral Fractures

As demonstrated in Table 3, for the development set, the models using images that included both vertebral bone and paravertebral muscle showed significantly better AUROC, accuracy, and precision values compared to those using bone-only images. Specifically, the bone-only images had an AUROC of 0.677 (95% CI 0.674-0.680) and accuracy of 0.669 (95% CI 0.665-0.673). In contrast, the images including both bone and muscle exhibited an AUROC of 0.739 (95% CI 0.737-0.741) and accuracy of 0.719 (95% CI 0.715-0.722; all *P*<.001). The fracture risk assessment tool (FRAX) model for major osteoporotic fracture and hip fracture showed lower AUROCs of 0.557 and 0.563, respectively, indicating a significantly better performance of our image model (all *P*<.001).

Similar trends were observed in the external validation set, where bone-only images resulted in an AUROC of 0.815 (95% CI 0.806-0.824) and accuracy of 0.754 (95% CI 0.752-0.756), while the combined bone and muscle images demonstrated an AUROC of 0.827 (95% CI 0.821-0.833) and accuracy of 0.812 (95% CI 0.798-0.826; all *P*<.001), though the specificity value was similar between the 2 groups. The FRAX model for major osteoporotic fracture and hip fracture had AUROCs of 0.810 and 0.780, respectively. Again, these results confirmed the superior predictive capability of our image-based model (all *P*<.001).

**Table 3:** Performance comparisons of image models in predicting vertebral fractures.

	Development set				External validation set		
	Bone only	Bone + muscle	*P* value	Bone only		Bone + muscle	*P* value
AUROC^a^ (95% CI)	0.677 (0.674-0.680)	0.739 (0.737-0.741)	<.001	0.815 (0.806-0.824)		0.827 (0.821-0.833)	.04
Accuracy (95% CI)	0.669 (0.665-0.673)	0.719 (0.715-0.722)	<.001	0.754 (0.752-0.756)		0.812 (0.798-0.826)	<.001
Sensitivity (95% CI)	0.746 (0.739-0.753)	0.761 (0.746-0.776)	.23	0.645 (0.613-0.677)		0.704 (0.675-0.733)	.054
Specificity (95% CI)	0.601 (0.586-0.616)	0.634 (0.625-0.643)	.002	0.844 (0.810-0.877)		0.855 (0.835-0.875)	.43

^a^AUROC: area under the receiver operating characteristic curve.

### Comparisons Between the Performances of Image and Clinical Models

**Figure 4:**
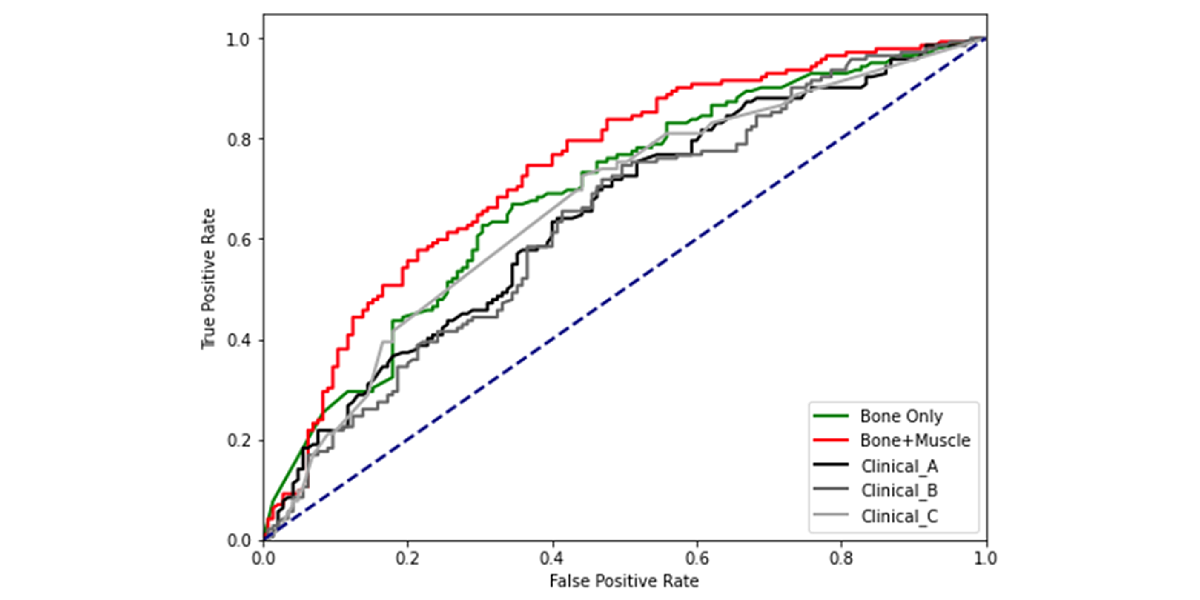
Receiver operating characteristic curves comparing models for 5-year vertebral fracture prediction: model A includes age and sex, model B additionally includes BMI, and model C additionally includes a history of drinking, smoking, and possible secondary osteoporosis.

Compared to the clinical models, the image model using vertebral bone and muscle showed significantly higher performance than the clinical models in predicting the vertebral fractures during the 5-year follow-up period in the development and external validation sets (Figure 4, Table 4). In the development set, the images that included vertebral bone and muscle had significantly better AUROC and accuracy than the clinical model D, which included age, sex, BMI, history of alcohol consumption, smoking, possible secondary osteoporosis, type 2 diabetes mellitus, HIV, hepatitis C infection status, and renal failure (AUROC 0.667, 95% CI 0.661-0.672 and accuracy 0.640, 95% CI 0.661-0.649; all *P*<.001, Table 4). In addition, the performance did not show a significant change when the clinical variables were added to the image-only model (Table S1 in Multimedia Appendix 1).

**Table 4:** Performance comparisons of image and clinical variable models in predicting fractures.

			AUROC^a^ (95% CI)		*P* value		Accuracy (95% CI)		*P* value		Sensitivity (95% CI)		*P* value	Specificity (95% CI)		*P* value
**Development set**																
	Image-only^b^	0.739 (0.737-0.741)		Reference		0.719 (0.716-0.722)		Reference		0.761 ± 0.024 (0.746-0.776)		Reference		0.634 (0.625-0.643)	Reference	
	Clinical model A^c^	0.647 (0.643-0.651)		<.001		0.620 (0.614-0.626)		<.001		0.681 (0.643-0.719)		.03		0.575 0.549-0.601)	<.001	
	Clinical model B^d^	0.631 (0.626-0.636)		<.001		0.612 (0.610-0.614)		<.001		0.675 (0.639-0.711)		.02		0.558 (0.517-0.598)	.003	
	Clinical model C^e^	0.663 (0.659-0.667)		<.001		0.637 (0.631-0.643)		<.001		0.723 (0.694-0.752)		.11		0.553 (0.521-0.585)	<.001	
	Clinical model D^f^	0.667 (0.661-0.672)		<.001		0.640 (0.661-0.649)		<.001		0.729 (0.690-0.768)		.13		0.560 (0.527-0.593)	.005	
	FRAX^g^ (MOF)^h^	0.557^i^		<.001		0.557^i^		<.001		0.442^i^		<.001		0.672^i^	<.001	
	FRAX (hip)	0.563^i^		<.001		0.556^i^		<.001		0.449^i^		<.001		0.663^i^	<.001	
**External validation set**																
	Image-only	0.827 (0.821-0.833)		Reference		0.812 (0.798-0.826)		Reference		0.704 (0.675-0.733)		Reference		0.855 (0.834-0.875)	Reference	
	Clinical model A	0.731 (0.725-0.737)		<.001		0.651 (0.629-0.673)		<.001		0.715 (0.683-0.747)		<.001		0.656 (0.629-0.683)	<.001	
	Clinical model B	0.733 (0.725-0.737)		<.001		0.654 (0.625-0.683)		<.001		0.728 (0.673-0.783)		<.001		0.662 (0.621-0.703)	<.001	
	Clinical model C	0.745 (0.733-0.757)		<.001		0.669 (0.646-0.692)		<.001		0.713 (0.678-0.748)		<.001		0.720 (0.689-0.751)	<.001	
	Clinical model D	0.749 (0.736-0.762)		<.001		0.675 (0.643-0.707)		<.001		0.729 (0.690-0.768)		<.001		0.686 (0.650-0.722)	<.001	
	FRAX (MOF)	0.810^i^		<.001		0.810^i^		<.001		0.262^i^		<.001		0.887^i^	<.001	
	FRAX (hip)	0.780^i^		<.001		0.685^i^		<.001		0.705^i^		<.001		0.682^i^	<.001	

^a^AUROC: area under the receiver operating characteristic curve.

^b^Image model represents the model using bone and muscle.

^c^Model A includes age and sex.

^d^Model B additionally includes BMI.

^e^Model C additionally includes history of drinking, smoking, and possible secondary osteoporosis.

^f^Model D includes age, sex, BMI, history of alcohol consumption, smoking, possible secondary osteoporosis, type 2 diabetes mellitus, HIV, hepatitis C infection status, and renal failure.

^g^FRAX: fracture risk assessment tool.

^h^MOF: major osteoporotic fracture.

^i^Since this was calculated for a single data set, there are no 95% CI values.

As depicted in Figure 4, in the external validation set, the images including vertebral bone and muscle showed a significantly better AUROC and accuracy than the clinical model D (AUROC 0.749, 95% CI 0.736-0.762 and accuracy 0.675, 95% CI 0.643-0.707; all *P*<.001). The results were similar for clinical models A, B, C, and D, which showed poorer performance than the image model.

## Discussion

In this study, we developed and externally validated a vertebral fracture prediction model by using abdominal CT images. In the development cohort, the performance of predicting vertebral fractures represented by AUROC was 0.688 (SD 0.001) by using images of vertebral bone-only and 0.736 (SD 0.003) by using images of vertebral bone and paravertebral muscle. In the validation cohort, the performances (AUROC) were 0.698 (SD 0.001) and 0.729 (SD 0.002) for images of vertebral bone-only and images of vertebral bone and paravertebral muscle, respectively. In addition, the performance of the model using images of vertebral bone and muscle was significantly better than that of the clinical models using age, sex, BMI, use of steroids, smoking status, and possible secondary osteoporosis, which showed performances of 0.635 (SD 0.002) and 0.698 (SD 0.021), respectively, for the development and validation cohorts.

Our model shows that the image models using vertebral bone and muscle had a better performance than those using images of vertebral bone-only. Osteosarcopenia, defined by combined occurrence of bone loss and sarcopenia, is one of the critical risk factors for osteoporotic fractures [25,26]. The paravertebral muscles are essential components of the vertebral column and are associated with osteoporotic vertebral fractures [27,28]. In previous studies, information retrieved from muscle images, such as cross-sectional area, volume, and degree of fat infiltration in the paravertebral muscle, was correlated with vertebral stability and the risk of fractures [28,29]. Specifically, Kim et al [30] reported lower cross-sectional areas and greater fat infiltration of the paravertebral muscles in patients with vertebral fractures than in those without fractures. This implies that not only the density and quality of the bones are correlated with the risk of fractures but also the quality of the muscles supporting and communicating with the bones [17]. Fat infiltration in the muscles, called myosteatosis, has been reported to be associated with an increased risk of fractures [17,31]. Thus, in line with previous studies, our study results imply that information from the images of the paravertebral muscles in addition to the information from the images of vertebral bones can help predict vertebral fractures more accurately.

Further, the image-based learning model with images of both vertebral bone and muscle showed better performance than the clinical variable–based models. This finding is consistent with a previous report that showed that information from the images of vertebral bones and muscles from CT scans can be used to predict major osteoporotic fractures and is comparable with FRAX [32]. Another group reported different algorithms by using opportunistic CT-based bone assessments for osteoporotic fracture prediction [33]. They showed that CT-based predictors (vertebral compression fractures, simulated DXA T-scores, and lumbar trabecular density) with metadata of age and sex showed better performance in AUROC than FRAX [33]. However, in that model, muscle information was not considered [33], which may further improve the performance. In addition to the attenuation information, we used information from the image itself on the quality of the bone and muscle structure, similar to the trabecular bone score [13]. The trabecular bone score is an algorithm used to calculate the microstructure of the bone based on DXA images [34]. More than 50% of the osteoporotic fractures occur in patients with a normal or osteopenic range of BMD [35], which implies that the microarchitecture of the bone is also a key determinant of bone strength [36]. Similarly, in our study, the model used the information on the qualities of bones and muscles from CT images, demonstrating the potential value of CT images that may include rich and various informative data for the metabolic diseases of bones and muscles.

We also observed that the performance did not significantly change when clinical variables were added to the image-only model. There is a possibility that information such as age and gender could already be reflected to some extent in the image itself [37]. Therefore, there could be an insignificant improvement in the performance because the information poses a redundant input to the model. It is widely accepted that there is a noticeable sex difference in the size of the vertebral body and paravertebral muscles [37], and BMI could be positively correlated with the size of the vertebrae and muscles. In addition, although the model was based on high-resolution peripheral quantitative CT, each bone has different characteristics according to age and sex, such as calcification and size, which could have influenced our analysis [38]. In addition, the vertebral endplate calcification increases with age, implying that age information can be reflected in the image [39]. In addition to that reported in previous studies, smoking and alcohol consumption status can be associated with low muscle mass [40], which may explain why adding simple clinical variables to the image may not significantly improve the model, as the image already contains some clinical information. The results are clinically promising, and they can be utilized in the future, as only opportunistic CT scans without detailed clinical variables may automatically provide the risk of osteoporotic fractures.

To extract pertinent information from each CT scan, we designed an image-only model to prevent overfitting and to focus on the essential regions. Since 3D CNN models, which have a large number of parameters to be optimized, tend to overfit the training data [41], the CNN encoder of our model took consecutive 2D images as its input data while keeping their sequential information with the RNN decoder [42]. The input processing strategy served as a robust data augmentation method because our model could exploit different 2D image sets from a single 3D CT scan at each training iteration. In addition, an attention module was applied to the CNN encoder to further enhance its robustness. The attention module automatically guided the image model to concentrate on essential regions [43] for the prediction of vertebral fractures. Thus, the attention CNN-RNN model avoids making predictions based on background regions, except for the vertebral body and paravertebral muscles. Unlike previous CNN model–based deep learning algorithms, which were limited to 2D X-ray analysis or bone texture analysis, our CNN-RNN model showed robust performance in fracture prediction. Owing to its design to mitigate the overfitting problem of conventional 3D CNN models [42], the CNN-RNN model could extract effective information from 3D CT images, which were intractable in previous approaches. In addition, the attention module forced our model to focus on important regions in the CT images by removing the effects of the background regions [43].

Our study has several limitations. The data set did not contain BMD due to the retrospective study design, which is an essential predictor for osteoporotic fracture. It was difficult to compare the clinical model containing BMD with the image model. The model showed a 5-year fracture prediction model instead of a 10-year model owing to the follow-up duration of the data set, which is relatively short to be utilized in real-world practice. Thus, due to the short time frame, we could not show the results for nonvertebral fractures because the number of cases was too small. In addition, the paravertebral muscles were included without distinction among the psoas, intervertebral, multifidus, longissimus, iliocostalis, and quadratus lumborum muscles. Therefore, it is difficult to interpret the contribution of each muscle. In addition, the number of images in the development set may not be sufficient for model optimization. Moreover, the utilization could be low in various contrast settings because it was based on contrast CT scans. There was also the disparity in vertebral fracture incidence between the development and the external validation set, which may affect the external validity and generalizability of our fracture prediction model. The retrospective nature inherently carries the potential for selection bias, including confounding by indication. Although we have employed propensity score adjustment to mitigate this bias, it is important to acknowledge that residual bias may still be present. Another limitation is the exclusion of radiographic imaging data with poor quality from our models. This decision might have introduced detection bias such that it may have impacted the diagnostic accuracy of our models in correctly identifying positive versus negative fracture cases. Further, we could not assess the reproducibility of these measurements through interexaminer and intraexaminer κ value assessments, which could be considered a limitation of our study. Future prospective studies could benefit from including such reproducibility assessments.

Our study has several strengths. Our study was longitudinally designed to observe future fracture events in patients who did not have baseline fractures. Furthermore, in the development cohort, we used controls with matched clinical variables, which made it possible to attenuate the effects of the major clinical variables in the model. It was also externally validated, which helped prove the generalizability of the model. In addition, the model used the image itself as an input, which made it possible to utilize the information on vertebral bone and muscle quality and quantity. This inclusion of the muscle image reflected the interplay between muscle health and fracture risk. For instance, factors such as muscle mass and muscle steatosis, which are visible in CT images as darker and more heterogeneous areas compared to normal muscle, could be crucial inputs. These muscle attributes, automatically analyzed by the CNN, contribute significantly to the model’s ability to discern patients at higher risk of fractures, offering a more comprehensive view than bone analysis alone. In addition, by sequentially applying bones and muscles to the model, it was possible to check the degree of contribution of muscles and bones to the model performance, thereby increasing the interpretability of the model. In addition, the differences in the clinical characteristics between development and external validation sets were purposefully leveraged to assess the generalizability of our model across populations with varying clinical profiles.

In this study, we showed that a deep learning model of the CNN-RNN structure based on CT images of the muscle and vertebral bone could help predict the risk of vertebral fractures. The model using images of the vertebral bone and muscle showed better performance than the model using images of the vertebral bone-only. This implies that the information from the muscle images provides additional key information for predicting fractures. In addition, the model using images showed better performance than the model using clinical variables, suggesting that images can provide useful information in addition to having known clinical variables. This study has clinical significance in suggesting that opportunistic CT screening with deep learning algorithms utilizing bone and muscle images may contribute to identifying patients with a high fracture risk in the future. Further prospective studies are needed to broaden the applicability of our model.

## Appendix

Multimedia Appendix 1 Supplementary table and figure.

## Abbreviations

AUROC: area under the receiver operating characteristic curve
BMD: bone mineral density
CNN: convolutional neural network
CT: computed tomography
DXA: dual-energy X-ray absorptiometry
FRAX: fracture risk assessment
RNN: recurrent neural network

## Data Availability

The data that support the findings of this study are available on request from the corresponding author. The data are not publicly available due to privacy or ethical restrictions.
