# Supplementary material for: A Computed Tomography–Based Fracture Prediction Model With Images of Vertebral Bones and Muscles by Employing Deep Learning: Development and Validation Study
Source: J Med Internet Res. 2024 Jul 12;26:e48535. doi: 10.2196/48535 (PMC11282387; doi:10.2196/48535)
Supplement: Multimedia Appendix 1 [file jmir_v26i1e48535_app1.docx]

**Table S1. Performance Comparisons of Image and Combination Model of Image and Clinical Variables**

|  | **AUROC** | ***p*** | **Accuracy** | ***p*** | **Sensitivity** | ***p*** | **Specificity** | ***p*** |
| --- | --- | --- | --- | --- | --- | --- | --- | --- |
| **Development set** |  |  |  |  |  |  |  |  |
| **Image-only** | 0.739  (0.737 - 0.741) | - | 0.719  (0.716 - 0.722) | - | 0.761  (0.746 - 0.776) | - | 0.634  (0.625 - 0.643) | - |
| **Image + model A** | 0.734  (0.729 - 0.739) | 0.417 | 0.708  (0.704 - 0.712) | 0.001 | 0.750  (0.730 - 0.770) | 0.562 | 0.642  (0.623 - 0.661) | 0.766 |
| **Image + model B** | 0.732  (0.726 - 0.738) | 0.251 | 0.715  (0.713 - 0.717) | 0.424 | 0.755  (0.738 - 0.772) | 0.764 | 0.629  (0.615 - 0.642) | 0.452 |
| **Image + model C** | 0.736  (0.734 - 0.738) | 0.108 | 0.720  (0.719 - 0.721) | 0.198 | 0.759  (0.745 - 0.773) | 0.361 | 0.630  (0.613 - 0.647) | 0.144 |
| **Image + model D** | 0.737  (0.733 - 0.741) | 0.077 | 0.718  (0.716 - 0.720) | 0.259 | 0.762  (0.750 - 0.774) | 0.391 | 0.632  (0.621 - 0.643) | 0.047 |
| **External validation set** | | | | | | | | |
| **Image-only** | 0.827  (0.821 - 0.833) | - | 0.812  (0.798 - 0.826) | - | 0.704  (0.675 - 0.733) | - | 0.855  (0.834 - 0.875) | - |
| **Image + model A** | 0.810  (0.803 - 0.816) | <0.001 | 0.788  (0.770 - 0.806) | 0.015 | 0.620  (0.595 - 0.645) | <0.001 | 0.795  (0.778 - 0.812) | <0.001 |
| **Image + model B** | 0.820  (0.812 - 0.828) | 0.009 | 0.802  (0.775 - 0.829) | 0.138 | 0.681  (0.649 - 0.713) | 0.215 | 0.810  (0.783 - 0.837) | 0.002 |
| **Image + model C** | 0.825  (0.820 - 0.830) | 0.407 | 0.805  (0.767 - 0.843) | 0.378 | 0.701  (0.681 - 0.721) | 0.477 | 0.840  (0.816 - 0.864) | 0.003 |
| **Image + model D** | 0.824  (0.817 - 0.831) | 0.315 | 0.813  (0.796 - 0.830) | 0.726 | 0.699  (0.680 - 0.718) | 0.574 | 0.849  (0.817 - 0.881) | 0.039 |

AUROC, the area under the receiver operator curve. None of them were significant compared to image-only model. Model A includes age and sex, model B additionally includes body mass index, model C additionally includes the use of steroid, history of drinking, smoking, and possible secondary osteoporosis, and model D additionally includes type 2 diabetes mellitus, HIV, and hepatitis C infection status, and renal failure.

**Figure S1. Input Images for (A) Bone-only and (B) Bone+Muscle Models**

**
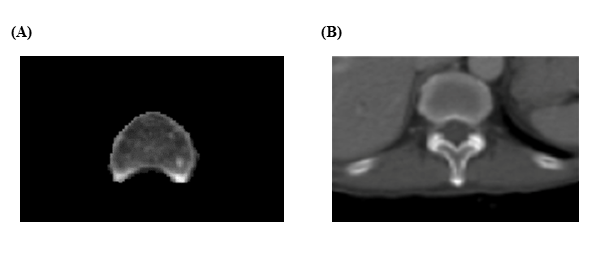
**
